# Editorial: Assisted dying in persons with mental illness

**DOI:** 10.3389/fpsyt.2022.1106384

**Published:** 2022-12-14

**Authors:** Manuel Trachsel, Anna Lisa Westermair, Marc Graf, Christian G. Huber

**Affiliations:** ^1^University Psychiatric Clinics (UPK) Basel, Basel, Switzerland; ^2^University Hospital Basel (USB), Basel, Switzerland; ^3^Institute of Biomedical Ethics and History of Medicine, University of Zurich (UZH), Zürich, Switzerland

**Keywords:** psychiatry, mental illness, assisted dying/suicide, ethics, autonomy, decision-making capacity, treatment resistance

Despite evidence-based treatment, some patients living with severe and persistent mental illnesses (SPMI) do not achieve a personally satisfying level of psychosocial wellbeing and functioning (1). In those patients with SPMI, quality of life is typically reported as low and life expectancy markedly shortened (2). Established treatments such as clozapine or electroconvulsive therapy (ECT), as well as newer approaches like ketamine or deep-brain stimulation (DBS) are available for treatment resistant symptoms but are not able to reach satisfying outcomes in all cases (3–6). Knowledge on how to best care for patients with SPMI is limited, both regarding empirical findings and accepted approaches or guidelines (7).

Some patients with SPMI may ask for access to assisted dying. Assisted dying encompasses different practices such as voluntary active euthanasia (VAE) or physician-assisted suicide. In VAE, the physician (or also nurse practitioner in Canada) administers the lethal drugs; in physician-assisted suicide, physicians or nurse practitioners (Canada) prescribe the lethal drugs, and the persons willing to die take them by themselves (8).

The legality of these forms of assisted dying varies considerably between jurisdictions. VAE is legal in Canada, the Netherlands, Belgium, and Luxembourg, with physician-assisted suicide being legal in those countries, too, and in many other countries including some states of the US, Colombia, and Switzerland (8). Assisted dying touches a wide variety of existential questions, basic values and worldviews about life and death, personal autonomy, liberty, stigma, suffering, and care for and protection of vulnerable persons. Assisted dying for people where SPMI is the sole underlying condition is particularly controversial (9), given long-standing questions such as when a mental health condition is presumed to be irremediable, if there is a distinction between health and mental health, as well as the long history of violence and abuse of power within the psychiatric profession. Further complicating the issue, psychiatry has the clinical and legally enforced duty to protect patients from illness-induced self-harm and suicide, at least partially conflicting with the idea of supporting or enabling assisted dying (10).

For this Special Topic, we have welcomed empirical, theoretical, ethics, and philosophical papers on assisted dying in SPMI. After peer-review, we have accepted five interesting, relevant and high-quality articles with different foci for publication.

The Research Topic starts with a conceptual analysis article by Zürcher on the notion of free will and the desire to die in mental illness. Zürcher begins with criticizing landmark court decisions and the practice of assisted dying organizations who highlight that persons with SPMI should only be eligible for assisted suicide if their death wish is not an expression of their illness. He argues that “it is irrelevant for the assessment of the desire to die whether it has been causally brought about by the mental illness”. What matters is an “internal reason that justifies the person's concern” which “must give expression to who the person essentially is and what the person fundamentally cares about”. Zürcher concludes that “a professional assessment of the desire to die of mentally ill persons must consist primarily in clarifying whether the desire to die fulfills the stated conditions for freedom, irrespective of the mental illness”.

The second contribution by Hachtel et al. is a policy review article on practical issues encountered by medical experts in the assessment of persons with mental disorders who ask for assisted dying in Switzerland. In particular, the authors discuss the current guidelines of the Swiss Academy of Medical Sciences (SAMS) and the Swiss Medical Association (FMH) regarding the difference between assisted dying requests by patients with somatic illness vs. patients with SPMI. Hachtel et al. conclude “that persons with a mental illness seem to face extra obstacles in relation with somatically ill persons as the assessment of the prerequisites comprises additional requirements.” The authors call for a scientifically guided elaboration of standards for mental healthcare professionals with regard to the contents and procedures in the assessment of assisted dying requests by patients with SPMI.

The third contribution by Kowalinski et al. describes a protocol for a survey study on physician-assisted dying in mentally and somatically ill individuals in Switzerland. According to the authors, one important reason why physician-assisted dying in persons with SPMI is such a controversial topic lies in the following conflicts: on the one hand, regarding assisted dying requests, persons with mental disorders should not be discriminated compared to somatically ill persons while at the same time, their specific vulnerability must be considered. “On the other hand, treating physicians must be protected in their ethical integrity and need security” when assessing assisted dying requests by SPMI patients. Kowalinski et al. plan a prospective survey-based study on the attitudes and opinions regarding assisted dying requests by patients with somatic illness vs. mental disorders in Switzerland. To this end, the authors will develop 48 case vignettes differing with regard to type of illness, availability of a therapeutic option, tolerability of suffering, and decision-making capacity. “The survey sample will comprise 10,000 Swiss residents of the general population from all three language regions (German, Italian, and French) as well as 10,000 medical professionals” working in seven states (cantons) of Switzerland. “Each participant will be randomly assigned a somatic terminal, a somatic non-terminal, and a mental non-terminal case vignette.”

As for the fourth contribution to this Special Topic, De Hert et al. comment on the improvement of control over voluntary active euthanasia (VAE) of persons with mental disorders. Belgium is one of the few jurisdictions which allow VAE. The Belgian Euthanasia Law knows a so-called three-level control system which is applied in the evaluation of VAE requests from persons who suffer unbearably from an SPMI. De Hert et al. use a recent lawsuit against three physicians as the starting point for their analysis of the adequacy of this system. They found that the patient in this trial “was euthanized without it having been substantiated that her psychiatric illness had no prospect of improvement and that her suffering could not be alleviated” and conclude that the three-level control system “appears to have failed at each level”. Consequently, a model for revising the Belgian Euthanasia Law is suggested which, in case of euthanasia requests from persons with SPMI, integrates the “advice of two psychiatrists, and face-to-face discussions between all physicians involved”. In parallel, they suggest that “a treatment track should be guaranteed where reasonable evidence-based treatments and recovery-oriented options are tried”.

Franke et al. provide the final contribution to this Special Topic, a literature review on ethical and legal issues about assisted dying requests from people in detention. The authors begin their article with pointing to the principle of equivalence of care, i.e., people in detention must have access to the same standards of healthcare services as all other people. According to Franke et al., this principle can be applied to assisted dying requests from persons with SPMI in detention, too. Based on this assumption, the authors discuss the issue that “detention itself can lead to psychological distress and suicidality, so we must consider whether and how people in such settings can make autonomous decisions”. In their article, Franke et al. “compare different practices for dealing with requests for assisted dying from people in prison and forensic psychiatric facilities and discuss the current ethical and psychiatric issues concerning assisted dying in such settings”. Thereby, the authors devote a special focus to the concepts of free will (see also the contribution of Zürcher in this Special Topic), the right to life, personal integrity, and the right of a state to inflict punishment.

The five articles in this Special Topic offer fascinating insights into the various issues in the field of assisted dying in SPMI. As editors, we hope that these contributions will inspire other scientists to plan and conduct more research in this still understudied but highly relevant field. Furthermore, we are convinced that this Research Topic also serves policy makers and professional committees to elaborate regulations and guidelines.

## Author contributions

MT wrote the first draft of the manuscript. CH, MG, and AW critically revised it. All authors read and approved the final version.
